# Phylogeny and Functional Traits Jointly Shape Global Rice Pest Invasions

**DOI:** 10.3390/insects17050500

**Published:** 2026-05-14

**Authors:** Jiayuan Xie, Yuan Yuan, Ziqi Chen, Liuxin Qiao, Jun Xu

**Affiliations:** 1 State Key Laboratory of Breeding Biotechnology and Sustainable Aquaculture (CAS), Institute of Hydrobiology, Chinese Academy of Sciences, Wuhan 430072, China; jyxie2023@gmail.com (J.X.);; 2 State Key Laboratory of Lake and Watershed Science for Water Security, Institute of Hydrobiology, Chinese Academy of Sciences, Wuhan 430072, China; 3 University of Chinese Academy of Sciences, Beijing 100049, China; 4 State Key Laboratory of Marine Resource Utilization in South China Sea, School of Ecology, School of Marine Biology and Fisheries, Hainan University, Haikou 570228, China

**Keywords:** invasion biology, crop protection, pest management, invasion mechanisms, phylogenetic relationships

## Abstract

Biological invasions by crop pests increasingly threaten global food security, yet the mechanisms underlying invasion success remain poorly understood, particularly within focal crop systems. Here, we examined how evolutionary history and species traits jointly shape rice pest invasions using a global dataset of 129 rice pests. We reconstructed a COI-based phylogeny, compiled ten functional traits, and analyzed invasion status using Bayesian phylogenetic and non-phylogenetic generalized linear mixed models. Invasive rice pests were non-randomly distributed across the phylogeny, with more than 50% of species in Aphididae, Gryllotalpidae, and Noctuidae being invasive. Most functional traits showed phylogenetic signal, whereas invasion status exhibited a weaker but still non-random phylogenetic pattern. Incorporating phylogeny improved model performance, with phylogenetic effects explaining more variation than measured traits alone. After accounting for phylogenetic dependence, fecundity remained the strongest positive predictor of invasion status, whereas host number, body length, and migration distance showed weaker directional trends. These findings show that rice pest invasions are jointly shaped by phylogenetic background and functional traits, and provide a basis for phylogeny-informed risk screening and biosecurity management.

## Simple Summary

Biological invasions by rice pests increasingly threaten global rice production, yet it remains unclear why some species become invasive while others do not. In this study, we examined 129 major rice pests worldwide to test whether becoming invasive is associated with evolutionary history and species traits. We found that invasive rice pests are not randomly distributed across groups of related species. Some lineages, especially aphids, mole crickets, and noctuid moths, exhibited higher proportions of invasive species than others. We also found that species with higher reproductive output were more likely to be invasive, while several other traits showed weaker patterns. Overall, our results show that rice pest invasions are shaped by both shared ancestry and species traits. These findings suggest that early warning and quarantine programs could be improved by prioritizing high-risk pest groups and species with high reproductive output.

## 1. Introduction

Owing to globalization and the resulting breakdown of biogeographic barriers, species have been introduced into new regions at an unprecedented rate since the last century [1]. Some of those alien species have been successfully naturalized in the new ranges, and a subset have become invasive, often causing biodiversity loss and the disruption of ecosystem services [2,3]. Insects comprise one of the largest taxonomic groups among invasive species, and many of them are major pests in agriculture and forestry [4]. Agricultural pests are a major constraint on global crop production and cause roughly 40% of annual crop losses [5]. In this context, invasive pests further intensify pest pressure and crop damage, and agriculture has incurred the highest economic costs among primary production sectors affected by biological invasions [6]. Despite their major agricultural and economic impacts, the mechanisms underlying the invasiveness of crop pests remain poorly understood.

Invasion success is often influenced by species traits related to growth, reproduction, and resource use, highlighting the importance of functional traits in explaining invasiveness [7,8,9,10,11]. So far, comparative studies of invasive and non-invasive insects have shown that invasive species are often associated with higher fecundity, smaller body size, broader habitat breadth, and lower thermal requirements [12,13,14,15]. However, evidence remains limited for crop pests, particularly within specific crop systems. Although several invasive crop pests, such as the whitefly (*Bemisia tabaci*), aphid and the Mediterranean fruit fly (*Ceratitis capitata*) have been intensively studied, comparative analyses of invasive and non-invasive pests within the same crop system remain scarce [13,14].

However, invasion mechanisms cannot always be adequately explained by functional traits alone, especially when trait data are incomplete or when important ecological differences are not directly measured. Under the phylogenetic niche conservatism hypothesis, closely related species tend to share similar ecological niches, adaptive strategies, and functional traits; accordingly, evolutionary history may strongly influence invasion success [16,17]. Incorporating phylogenetic information has been shown to significantly improve model performance in the study of invasion success [18,19]. Moreover, phylogeny can capture unmeasured biological differences among species and provide a basis for imputing missing trait values in global functional datasets [20,21,22]. Together, these considerations highlight the need for an integrated phylogeny–trait framework to better understand the mechanisms of biological invasions.

Rice (*Oryza sativa* L.) is one of the world’s most important staple crops, providing food for more than half of the global population [23]. However, rice production is threatened by hundreds of arthropod pests, many of which have invaded new regions and caused substantial yield losses [24,25,26]. Previous studies have evaluated the potential invasion risk for several important rice pests, such as *Spodoptera frugiperda*, *Steneotarsonemus spinki*, and *Lissorhoptrus oryzophilus*, primarily using species distribution models [27,28,29]. Although these studies have provided valuable species-specific insights, there is still a lack of analysis of the underlying mechanisms of rice pest invasion success driven by functional traits and phylogenetic relationships [14].

To address this gap, we integrated invasion records, phylogenetic relationships and functional traits for 129 major rice pests globally. Using Bayesian regression frameworks, we evaluated the effects of phylogenetic relationships and functional traits on the invasion status of rice pests to clarify the mechanisms underlying rice pest invasions. Based on previous evidence that invasiveness can be phylogenetically structured and associated with species traits [12,13,14,18,19], we hypothesized that (1) the invasion status of rice pests is phylogenetically clustered, with some clades containing disproportionately more invasive species; (2) the phylogenetic relationship contributes significantly to explaining invasion status of rice pests; and (3) functional traits retain explanatory power after accounting for phylogenetic dependence. Our study aims to enhance the understanding of the underlying mechanisms driving rice pest invasions and to provide a phylogeny–trait basis for future early invasion risk assessments.

## 2. Materials and Methods

### 2.1. Species List and Invasion Records

We compiled a checklist of global major rice pests in July 2024. First, we extracted candidate species from online databases, including CABI CPC [25], InvaCost [30] and the International Rice Research Institute Knowledge Bank. The checklist was further supplemented using monographs as well as scientific and gray literature [24,31,32,33]. We then validated the scientific names of all candidate species against the Global Biodiversity Information Facility (GBIF) and the CABI CPC [25], retaining the accepted names and resolving synonyms. Following CABI guidance, candidate species were screened according to their documented geographic distribution and economic impacts. We retained only species repeatedly reported as major pests of rice and excluded minor pests or species with only strictly localized impacts [25]. The final dataset comprised 129 species, hereafter rice pests, spanning seven orders and classified into seven feeding guilds (Supplementary Table S1). Invasion status was assigned based on a literature review. Species with documented records of establishing self-sustaining populations outside their native ranges were classified as invasive, whereas species without documented invasion records were treated as non-invasive for analytical purposes.

### 2.2. Phylogenetic Tree

**Figure 1:**
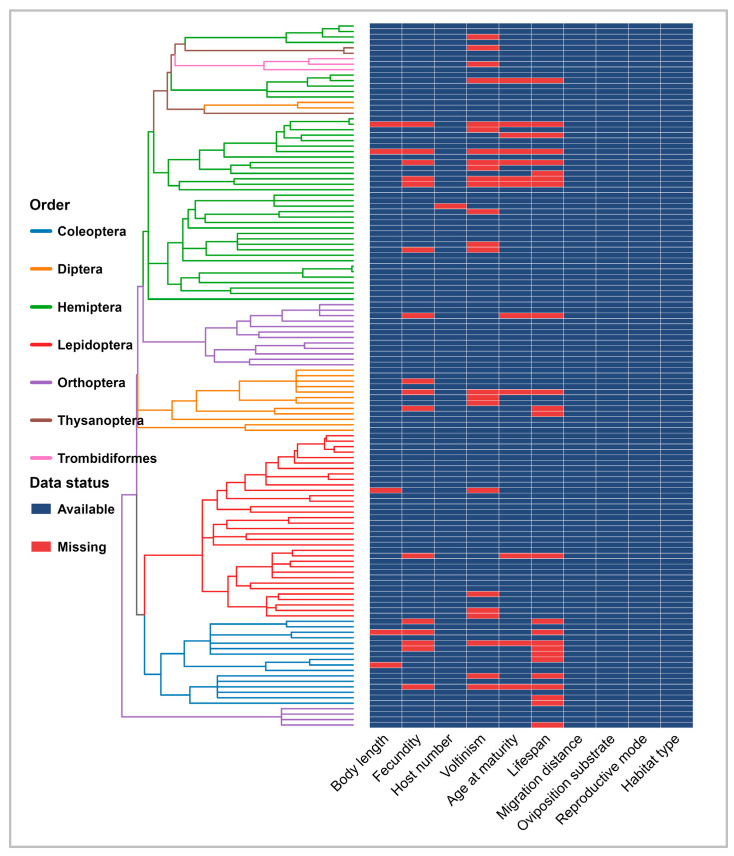
The phylogenetic relationships of rice pests and distribution of missing data of functional traits. The left panel shows the phylogenetic tree constructed based on COI sequences, with branch colors representing the orders of the pests. The right heatmap displays the coverage of functional trait data for each species, where blue indicates available data and red indicates missing values. Missing continuous traits were later estimated using the phylogenetic imputation procedure described in Section 2.4.

To reconstruct the phylogenetic tree of global rice pests, we retrieved cytochrome c oxidase subunit I (COI) sequences from NCBI GenBank and the BOLD Systems database using the rentrez and bold packages [34,35]. Because multiple sequences or complete mitochondrial genomes were available for some species, we applied a sequence selection strategy to retain the most informative and highest-quality COI record for each species. For species represented by multiple sequences, COI regions from complete mitochondrial genomes were prioritized; otherwise, the most complete and highest-quality fragment was selected. Sequences were aligned in MAFFT using the automatic strategy, and the resulting alignments were trimmed in TrimAl using the automated1 mode to remove poorly aligned regions [36,37]. Phylogenetic inference was then conducted under maximum likelihood (ML) in IQ-TREE2 using the GTR+G model with 1000 ultrafast bootstrap replicates [38]. Divergence times were subsequently estimated by penalized likelihood using the ape package [39]. Among the 129 species included in this study, 91 were represented by available COI sequences and were incorporated directly into the initial phylogenetic tree, whereas 38 species lacked sequence data (Supplementary Table S1). To retain as many species as possible in the comparative analyses, these missing species were subsequently inserted into the tree according to their taxonomic affinities using the phytools package (Figure 1) [40]. Specifically, species were placed relative to their nearest available relatives, beginning at the genus level and moving upward when necessary, with branch lengths assigned from mean phylogenetic distances.

### 2.3. Functional Trait Data

We compiled a functional trait dataset for the 129 rice pest species, including 10 traits related to growth, reproduction, host use, and habitat characteristics (Supplementary Table S2). The dataset comprised seven continuous traits (body length, fecundity, host number, voltinism, age at maturity, lifespan, migration distance) and three categorical traits (oviposition substrate, reproductive mode, and habitat type), assembled from published articles, academic monographs, and online sources. Migration distance was treated as a semi-quantitative continuous trait, based on observed values when available and otherwise conservative estimates based on movement mode and body size. For the categorical traits, missing values were supplemented using the lowest available taxonomic level, prioritizing species-level information, followed by genus- and family-level information when necessary, as these traits are relatively conserved among close relatives [41,42]. Overall, 9.08% of trait values were missing, with trait-specific missingness ranging from 0% for host number to 18.6% for voltinism and lifespan (Figure 1). Missing categorical traits were supplemented using the lowest available taxonomic level, whereas missing continuous traits were estimated using the phylogenetically informed imputation procedure described in Section 2.4.

### 2.4. Phylogenetic Signal and Trait Imputation

To assess the phylogenetic conservatism of invasion status and functional traits, we quantified phylogenetic signal using different metrics for continuous and categorical variables. Continuous traits were evaluated using Pagel’s λ and Blomberg’s K using phytools package [40]. For Pagel’s λ, values close to 1 indicate strong phylogenetic dependence, whereas values near 0 indicate weak phylogenetic structure [43]. For Blomberg’s K, values close to 1 are consistent with a Brownian motion expectation, values greater than 1 indicate stronger phylogenetic conservatism than expected under Brownian motion, and values below 1 indicate weaker phylogenetic signal [44]. Categorical variables, including invasion status, were assessed using Fritz and Purvis’s D via caper package [45]. Where D = 0 corresponds to a Brownian phylogenetic pattern, D = 1 indicates a random distribution, D < 0 indicates stronger phylogenetic conservatism than expected under Brownian motion, and D > 1 indicates phylogenetic overdispersion [46]. The significance of D was evaluated against both Brownian and random null expectations: (i) *p*Brownian (D < 0 indicates greater conservatism than Brownian expectation), and (ii) *p*Random (D > 1 indicates overdispersion).

When functional traits are phylogenetically conserved, phylogenetic imputation is a valuable approach for estimating missing functional traits [20,47]. Prior to imputation, all continuous traits were natural log-transformed to improve distributional properties and to avoid biologically implausible negative values on the original scale. We statistically imputed missing values for continuous traits with phylogenetically informed predictive models using the phylopars package [47]. We then compared alternative evolutionary models of trait covariance, including Brownian motion, Pagel’s λ, Ornstein–Uhlenbeck, and Early-Burst models, and selected the best-fitting model based on Akaike’s Information Criterion (Supplementary Table S3). Finally, we selected the best-performing model (Pagel’s λ) to impute missing continuous values (Supplementary Table S4).

### 2.5. Statistical Analysis

We quantified the effects of functional traits on invasion status while accounting for phylogenetic dependence. To do so, we fitted a Bayesian phylogenetic generalized linear mixed model (PGLMM) with a Bernoulli error distribution and logit link function using the brms package in R [48]. Functional traits were included as fixed effects, whereas the phylogenetic relatedness and feeding guilds were included as random effects, with phylogenetic relatedness represented by a phylogenetic covariance matrix. Prior to modeling, we assessed multicollinearity among predictors using Spearman’s correlation coefficients and variance inflation factors (VIFs), and then selected predictors for models. The model was defined as follows:

(1) $$\mathrm{logit}\left(P_{j}\right)=\alpha +\sum_{i=1}^{n}\beta_{i}X_{ij}+\mu_{phylo(j)}+\mu_{feeding(j)}$$

where $P_{j}$ is the probability that species j is invasive, α is the intercept, $\beta_{i}$ represents the coefficients of trait i, $X_{ij}$ is the value of trait i for species j, and $\mu_{phylo(j)}$ and $\mu_{feeding(j)}$ are random intercepts associated with phylogeny and feeding guilds, respectively. Weakly informative priors (mean = 0; SD = 0.5) were assigned to the regression coefficients. Model parameters were estimated from four Markov Chain Monte Carlo (MCMC) chains, each with 6000 iterations, with the first 4000 iterations discarded as warmup [48]. Model convergence was assessed based on stable MCMC trace plots, R-hat values close to 1.0, and adequate bulk and tail effective sample sizes (Supplementary Figure S1, Table S5). To evaluate the contribution of phylogenetic structure to model performance, we additionally fitted a Bayesian generalized linear mixed model (GLMM) with the same fixed effects but without the phylogenetic random effect. Variance decomposition was used to compare the relative contributions of fixed effects and random effects to variation in invasion status between the Bayesian PGLMM and Bayesian GLMM. Because variable importance evaluation is not directly available for the Bayesian regression framework, we also fitted a standard GLMM using lme4 package and used hierarchical partitioning implemented in the glmm.hp package as an auxiliary analysis to estimate the relative contribution of each trait [49,50]. All analyses and visualization were performed in R version 4.3.3 [51].

## 3. Results

### 3.1. Invasion Status of Global Rice Pests

**Figure 2:**
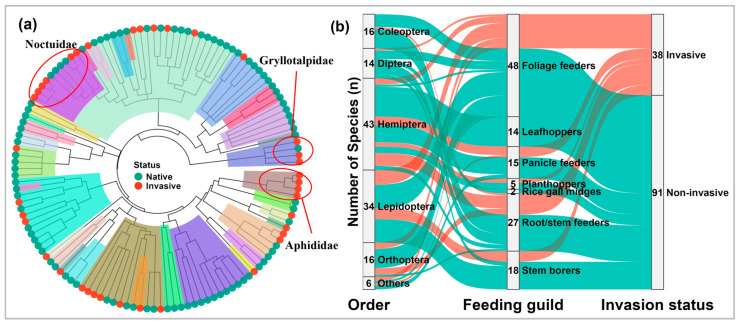
The composition and invasion status of global rice pests. (**a**) A phylogenetic tree and species invasion status; branches of different colors represent different families. The highlighted three families showed invasion proportions above 50%, whereas other families had lower proportions or no documented invasive species. (**b**) The composition of rice pests across taxonomic orders, feeding guilds, and invasion status; node height represents the number of species.

Among the 129 rice pest species included in this study, 38 species (29.5%) had documented invasion records, whereas 91 species (70.5%) had no documented invasion records (Figure 2). Invasive species were unevenly distributed across taxonomic groups. At the family level, more than 50% of species in Aphididae, Gryllotalpidae, and Noctuidae were invasive, whereas none of the 11 species in Cicadellidae had documented invasion records (Figure 2a, Supplementary Table S1). At the order level, most invasive pests (86.8%) belonged to Hemiptera, Lepidoptera, and Orthoptera, with Hemiptera containing the largest number of invasive species (n = 15, 39.5% of all invasive species) (Figure 2b, Supplementary Figure S2). However, the number of species in an order did not strictly correspond to its invasion proportion. For example, Trombidiformes had the highest invasion proportion (66.7%), despite being represented by only three species, whereas Diptera and Coleoptera showed comparatively low invasion rates (both <8%). Patterns also varied among feeding guilds. Foliage feeders contained the largest number of invasive species (n = 16, 42.1% of all invasive pests), followed by root/stem feeders (n = 10) (Figure 2b, Supplementary Figure S2). In contrast, planthoppers had the highest invasion proportion (40%), followed by root/stem feeders (37%). By comparison, all species were non-invasive among leafhoppers or rice gall midges, despite these two guilds together comprising 16 species.

### 3.2. Phylogenetic Signals of Functional Traits and Invasion Status

Most functional traits of global rice pests showed clear phylogenetic signal, whereas invasion status exhibited a weaker but still non-random phylogenetic distribution (Table 1). Among continuous traits, body length (K = 2.221, λ = 1.002), lifespan (K = 2.004, λ = 1.006), and age at maturity (K = 1.598, λ = 1.005) showed the strongest phylogenetic signals with *p*Random < 0.001. Fecundity (K = 0.538, λ = 0.875), voltinism (K = 0.650, λ = 0.837), and migration distance (K = 0.582, λ = 0.547) also exhibited a significant, albeit weaker, phylogenetic structure (all *p*Random < 0.003). Host number showed no significant signal based on Blomberg’s K (K = 0.418, *p*Random = 0.14), whereas Pagel’s λ remained significant (λ = 0.342, *p*Random < 0.001), suggesting weak but detectable phylogenetic structure. Among categorical traits, oviposition substrate, reproductive mode, and habitat type all showed strong phylogenetic conservatism, with D values ranging from −1.896 to −0.941. These traits differed markedly from a random distribution (all *p*Random < 0.001) and were broadly consistent with a Brownian expectation (all *p*Brownian > 0.973). Furthermore, invasion status exhibited a non-random phylogenetic structure (D = 0.354, *p*Random < 0.001, *p*Brownian = 0.121), although its signal was weaker than that of the functional traits and remained broadly consistent with a Brownian expectation.

### 3.3. The Relationship Between Invasion Status and Functional Traits

**Figure 3:**
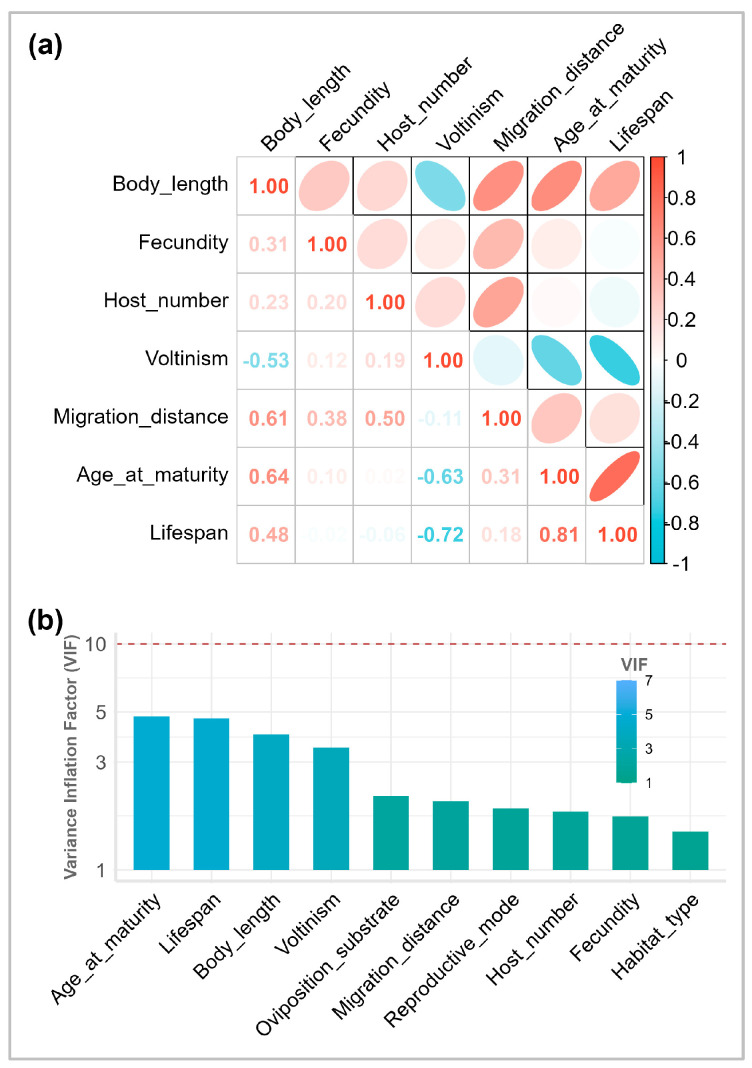
(**a**) Spearman correlation coefficients and (**b**) variance inflation factors among functional traits.

**Figure 4:**
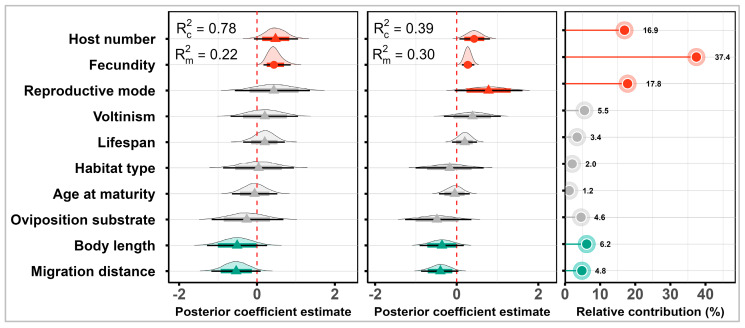
The relationships between functional traits and the invasion status of rice pests. In the left panel, the estimated posterior distributions are derived from the Bayesian PGLMM. In the middle panel, the estimated posterior distributions are derived from the Bayesian GLMM. Points represent posterior means; thick and thin horizontal bars indicate the 80% and 95% confidence intervals (CIs), respectively. The vertical dashed line at zero represents no effect. The conditional $R_{c}^{2}$ (based on both fixed and random effects) and $R_{m}^{2}$ (based fixed effects) for invasion status are reported in the Supplementary Table S5. The right panel shows the relative contribution of each factor to model performance. Obvious trends (80% CIs exclude zero) and significant effects (95% CIs exclude zero) are highlighted in red (positive effect) and green (negative effect).

Although age at maturity and lifespan were strongly correlated (|r| = 0.81), all traits had VIF values below 5 and were therefore retained for modeling [52] (Figure 3). Incorporating phylogenetic information substantially improved model performance. Variance decomposition showed that the Bayesian PGLMM had much higher overall explanatory power ($R_{c}^{2}\;$ = 0.78) than the Bayesian GLMM ($R_{c}^{2}$ = 0.39) (Figure 4). Accounting for phylogenetic dependence reduced the variance attributed solely to fixed effects of functional traits, with the marginal $R^{2}$ dropping from 0.30 (95% CI: 0.14–0.47) in the GLMM to 0.22 (95% CI: 0.06–0.39) in the PGLMM (Supplementary Table S6). Within the PGLMM framework, phylogenetic random effects accounted for the largest share of explained variation (0.48, 95% CIs: 0.13–0.82), explicitly exceeding both the fixed effect of functional traits (0.22, 95% CIs: 0.06–0.39) and the random effects of feeding guilds (0.09, 95% CIs: 0.00–0.41).

After accounting for phylogenetic dependence (Bayesian PGLMM), fecundity emerged as the most robust predictor of invasion status, exhibiting a strong positive effect (Figure 4, Supplementary Table S5). Host number showed a positive trend, whereas body length and migration distance exhibited negative trends; however, these effects were supported only at the 80% credible intervals. Notably, when phylogenetic relationships were ignored (Bayesian GLMM), the effects of host number and reproductive mode were inflated, both appearing as stronger positive associations. Consistent with these coefficient estimates, hierarchical variance partitioning based on the standard GLMM indicated that fecundity was the dominant functional predictor, accounting for 37.4% of the explained variance among traits (Figure 4). Reproductive mode (17.8%) and host number (16.9%) ranked next, whereas each of the remaining traits contributed less than 7%. This auxiliary analysis should be interpreted as a non-phylogenetic ranking of trait importance, rather than as a substitute for inference from the Bayesian PGLMM.

## 4. Discussion

Examining phylogenetic relatedness and functional traits together can improve understanding of invasion mechanisms in crop pests. Here, we show that global rice pest invasions are jointly shaped by phylogenetic background and functional traits. Invasive rice pests were phylogenetically clustered, indicating that invasion risk is unevenly distributed across lineages, whereas fecundity remained the strongest functional predictor after accounting for phylogenetic dependence. Thus, phylogeny and functional traits provide complementary explanations for rice pest invasiveness.

### 4.1. Phylogenetic Clustering of Invasive Rice Pests

Invasion status in rice pests was not randomly distributed across the phylogeny, but showed clear phylogenetic clustering, supporting our first hypothesis. This pattern is consistent with the broader view in invasion biology that invasiveness is phylogenetically structured [53]. Closely related species often share similar ecological niches, resource use, and life history strategies, and may therefore exhibit comparable probabilities of success across multiple invasion stages [22,53,54]. In our study, invasive species were concentrated in several lineages, particularly within Hemiptera, Lepidoptera, and Orthoptera, indicating that evolutionary history contributes to differences in invasion propensity among clades. At the same time, the phylogenetic signal of invasion status was weaker than that of most functional traits, suggesting that invasion risk is evolutionarily structured but not strictly conserved [53].

Several results further support this interpretation and our second hypothesis. Most functional traits exhibited clear phylogenetic signal, indicating that trait variation is strongly shaped by evolutionary history rather than randomly distributed across species [54,55]. The lower marginal R2 in the Bayesian PGLMM than in the Bayesian GLMM further suggests that phylogenetic relatedness accounted for part of the variation that would otherwise have been attributed solely to functional traits [56]. In addition, the phylogenetic random effect explained a larger share of variation than the measured functional traits, and the PGLMM showed substantially greater explanatory power than the non-phylogenetic GLMM. Together, these results suggest that phylogeny captures latent ecological and biological differences among species, such as physiological tolerances, behavioral plasticity, host associations, or dispersal opportunities, that were not fully represented in our trait dataset [22,57]. Therefore, incorporating phylogeny can help identify high-risk lineages and provide a broader framework for trait-based risk assessment [58].

### 4.2. Functional Trait Filtering of Invasive Rice Pests

Although phylogenetic relatedness explained most of the variation in invasion status, functional traits still retained explanatory power after controlling for phylogenetic dependence and feeding guild, supporting our third hypothesis. This suggests that functional traits do not act independently of evolutionary history, but instead further filter invasion outcomes within a phylogenetically constrained framework [53,59]. Relative to the Bayesian GLMM, the fixed effects of functional traits declined markedly in the Bayesian PGLMM, and the estimated effects of some traits, such as reproductive mode, were weakened after phylogenetic structure was incorporated. This pattern suggests that some trait effects detected in non-phylogenetic models may partly reflect similarities among related species, rather than fully independent ecological effects [56]. Thus, phylogenetic background may provide a baseline invasion tendency, whereas functional traits further influence whether species can successfully establish and expand [12,59].

Among all traits, fecundity was the strongest and most robust predictor of invasion status in both the Bayesian GLMM and PGLMM. Previous studies have shown that propagule pressure is a major determinant of invasion success, because the number of introduced individuals strongly influences whether populations can overcome demographic constraints during the early stages of invasion [60,61]. However, most agricultural pests are introduced unintentionally through global trades in commodities and plant materials, a pathway that may often involve relatively small initial propagule sizes [60,62]. Under such conditions, high fecundity could facilitate rapid population growth after introduction and thereby compensate, at least in part, for low initial propagule size, reducing extinction risks associated with small populations, environmental stochasticity, and genetic bottlenecks in early stages of invasion [63,64]. In addition, because many pest species are small-bodied and may remain undetected for some time after establishment, highly fecund lineages are more likely to reach outbreak densities and cause noticeable economic damage, thereby increasing the probability of being detected and recorded as invasive species [14,65,66].

By contrast, the effects of several other traits were weaker and were expressed mainly as directional trends rather than well-supported associations. Host number showed a positive trend, suggesting that polyphagous pests may be more likely to find suitable resources in novel environments and thus have a greater probability of establishment [67]. Body length exhibited a negative trend, which may relate to the introduction stage of invasion, as pests with smaller body size could be more easily transported with commodities and may also be less likely to be detected during quarantine inspections [68,69]. Migration distance, however, showed a weak negative trend, apparently contrasting with the common expectation that stronger dispersal should promote invasiveness [70,71]. One possible explanation is that our response variable is more closely related to successful introduction and establishment, whereas dispersal ability may be more important for post-establishment spread [2]. Additionally, highly mobile species may be less likely to remain associated with commodities during transport [72,73]. Although the remaining traits showed no clear associations with invasion status, they may still play important roles in pest invasion, but their effects may be contingent on invasion stage or phylogenetic background [18,74].

### 4.3. Implications for Pest Management

Effective management of rice pest invasions requires a shift from reactive eradication to proactive screening based on intrinsic biological predictors [75]. Given that invasion risk was concentrated in particular lineages, plant protection agencies may benefit from prioritizing surveillance of species closely related to known invasive pests, especially within clades showing elevated invasion propensity, such as Aphididae, Gryllotalpidae and Noctuidae. This phylogenetic perspective is particularly useful for early warning, because closely related species may share ecological tolerances and other latent characteristics that facilitate establishment [76,77].

Within such high-risk lineages, our results suggest that fecundity should receive particular attention, as highly fecund species may be better able to overcome demographic constraints during the early stages of invasion [60,78]. By contrast, traits such as broader host range or smaller body size may be better treated as supplementary indicators, because their effects in our analyses were weaker and primarily directional [72,79]. This hierarchical framework, using phylogeny to identify high-risk clades and functional traits to refine species-level priorities, could help agricultural biosecurity programs allocate monitoring and quarantine resources more efficiently.

### 4.4. Limitations

Despite providing new insights into rice pest invasion, this study has several limitations that should be acknowledged. First, invasion was simplified as a binary status, whereas biological invasions are inherently multi-stage processes involving introduction, establishment, and spread [2,80]. Moreover, species lacking documented invasion records were classified as non-invasive, which may introduce false negative bias because reporting effort is uneven across regions and taxa [65]. Second, both the phylogenetic and trait data are relatively coarse. Our phylogenetic tree was based primarily on the COI gene, with some species inserted according to taxonomic affinity, which may reduce the precision of phylogenetic covariance estimation. Likewise, some functional traits were imputed with phylogenetic imputation and migration distance was treated as a semi-quantitative variable, potentially weakening estimated associations with invasion status. Third, although the Bayesian regression framework identifies robust associations among phylogeny, functional traits, and invasion status, it does not by itself resolve the underlying causal mechanisms [81,82]. Future work would benefit from integrating finer functional traits and more explicit invasion stage information, higher-resolution phylogenies, and causal inference approaches. Nevertheless, because most studies of crop pest invasions focus on single species or distribution-based prediction, our study provides an important step toward a community-level understanding of pest invasion mechanisms within a focal crop system [29,83,84].

## 5. Conclusions

Biological invasions by rice pests represent an increasing threat to global rice production and agricultural biosecurity. Here, we show that invasion success in rice pests is not randomly distributed across the phylogeny, but is concentrated in particular evolutionary lineages. Incorporating phylogenetic relationships substantially improved model performance, indicating that shared evolutionary history captures important biological differences that are not fully represented by measured functional traits. Nevertheless, functional traits still contributed to invasion risk, with fecundity emerging as the most robust positive predictor after accounting for phylogenetic dependence. Other traits, including host number, body length and migration distance, showed weaker and more context-dependent associations. These findings suggest that rice pest invasions are jointly shaped by phylogenetic background and trait-based filtering. Thus, risk screening and quarantine prioritization may be improved by first identifying high-risk lineages and then refining species-level assessments using key traits such as reproductive output. More broadly, our study provides a phylogeny-informed framework for understanding and anticipating biological invasions in crop pest systems.

## Figures and Tables

**Table 1:** Phylogenetic signal of rice pest functional traits.

Functional Traits	Phylogenetic Signal	*p*Brownian	*p*Random
Body length	K = 2.221		0.001
	λ = 1.002		<0.001
Fecundity	K = 0.538		0.003
	λ = 0.875		<0.001
Host number	K = 0.418		0.14
	λ = 0.342		0.001
Voltinism	K = 0.65		0.001
	λ = 0.837		<0.001
Age at maturity	K = 1.598		0.001
	λ = 1.005		<0.001
Lifespan	K = 2.004		0.001
	λ = 1.006		<0.001
Migration distance	K = 0.582		0.001
	λ = 0.547		<0.001
Oviposition substrate	D = −0.971	0.993	<0.001
Reproductive mode	D = −0.941	0.973	<0.001
Habitat type	D = −1.896	1	<0.001
Invasion status	D = 0.354	0.121	<0.001

## Data Availability

The original contributions presented in this study are included in the article/Supplementary Material. Further inquiries can be directed to the corresponding author.

## Supplementary Materials

The following supporting information can be downloaded at: https://www.mdpi.com/article/10.3390/insects17050500/s1, Table S1. List of global major rice pests, invasion status and COI sequence accession numbers; Table S2. The composition of the functional trait dataset of rice pests; Table S3. Akaike’s Information Criterion of models for phylogenetic imputation; Table S4. The functional trait dataset of global major rice pests; Table S5. Parameter estimates for the Bayesian regression models; Table S6. Comparison of variance components between Bayesian PGLMM and Bayesian GLMM; Figure S1. MCMC sampling trajectory plot for fixed effects parameters in the Bayesian Models; Figure S2. Taxonomic and feeding guild patterns of invasive rice pests.
