# Supplementary material for: Phylogeny and Functional Traits Jointly Shape Global Rice Pest Invasions
Source: Insects. 2026 May 14;17(5):500. doi: 10.3390/insects17050500 (PMC13207550; doi:10.3390/insects17050500)
Supplement: Supplementary file 1 [file insects-17-00500-s001.zip › insects-4303198-supplementary.pdf]

# Supporting Information

## Table captions

Table S1. List of global major rice pests, invasion status and COI sequence accession numbers..

Table S2. The composition of the functional trait dataset of rice pests.

Table S3. Akaike's Information Criterion of models for phylogenetic imputation

Table S4. The functional trait dataset of global major rice pests.

Table S5. Parameter estimates for the Bayesian regression models.

Table S6. Comparison of variance components between Bayesian PGLMM and Bayesian GLMM.

## Figure captions

Figure S1 MCMC sampling trajectory plot for fixed effects parameters in the Bayesian Models.

Figure S2 Taxonomic and feeding guild patterns of invasive rice pests.

# Supporting Tables

**Table S1. List of global major rice pests, invasion status and COI sequence accession numbers.**

| ID | Class | Order | Family | Genus | Species | Feeder type | Invasion status | Accession Numbers |
|---|---|---|---|---|---|---|---|---|
| 1 | Arachnida | Trombidiformes | Tarsonemidae | *Steneotarsonemus* | *Steneotarsonemus spinki* | panicle feeders | Invasive | OM720121 |
| 2 | Arachnida | Trombidiformes | Tetranychidae | *Oligonychus* | *Oligonychus oryzae* | foliage feeders | Non-invasive | / |
| 3 | Arachnida | Trombidiformes | Tetranychidae | *Oligonychus* | *Oligonychus pratensis* | foliage feeders | Invasive | / |
| 4 | Insecta | Coleoptera | Brachyceridae | *Afroryzophilus* | *Afroryzophilus djibai* | root/stem feeders | Non-invasive | / |
| 5 | Insecta | Coleoptera | Brachyceridae | *Echinocnemus* | *Echinocnemus oryzae* | root/stem feeders | Non-invasive | / |
| 6 | Insecta | Coleoptera | Brachyceridae | *Echinocnemus* | *Echinocnemus squameus* | root/stem feeders | Non-invasive | OM772948 |
| 7 | Insecta | Coleoptera | Brachyceridae | *Hydronomidius* | *Hydronomidius molitor* | root/stem feeders | Non-invasive | / |
| 8 | Insecta | Coleoptera | Brachyceridae | *Lissorhoptrus* | *Lissorhoptrus brevirostris* | root/stem feeders | Non-invasive | / |
| 9 | Insecta | Coleoptera | Brachyceridae | *Lissorhoptrus* | *Lissorhoptrus oryzophilus* | root/stem feeders | Invasive | MW732716 |
| 10 | Insecta | Coleoptera | Brachyceridae | *Oryzophagus* | *Oryzophagus oryzae* | root/stem feeders | Non-invasive | / |
| 11 | Insecta | Coleoptera | Chrysomelidae | *Chaetocnema* | *Chaetocnema pulla* | foliage feeders | Non-invasive | / |
| 12 | Insecta | Coleoptera | Chrysomelidae | *Dicladispa* | *Dicladispa armigera* | foliage feeders | Non-invasive | KY845676 |
| 13 | Insecta | Coleoptera | Chrysomelidae | *Leptispa* | *Leptispa pygmaea* | foliage feeders | Non-invasive | KC427085 |
| 14 | Insecta | Coleoptera | Chrysomelidae | *Oulema* | *Oulema oryzae* | foliage feeders | Non-invasive | / |
| 15 | Insecta | Coleoptera | Chrysomelidae | *Trichispa* | *Trichispa sericea* | foliage feeders | Non-invasive | / |

| ID | Class | Order | Family | Genus | Species | Feeder type | Invasion status | Accession Numbers |
|---|---|---|---|---|---|---|---|---|
| 16 | Insecta | Coleoptera | Coccinellidae | *Chnootriba* | *Chnootriba similis* | foliage feeders | Non-invasive | KINS1656-11 |
| 17 | Insecta | Coleoptera | Scarabaeidae | *Holotrichia* | *Holotrichia serrata* | root/stem feeders | Non-invasive | KF939010 |
| 18 | Insecta | Coleoptera | Scarabaeidae | *Leucopholis* | *Leucopholis irrorata* | root/stem feeders | Non-invasive | / |
| 19 | Insecta | Coleoptera | Scarabaeidae | *Leucopholis* | *Leucopholis lepidophora* | root/stem feeders | Non-invasive | MH712758 |
| 20 | Insecta | Diptera | Cecidomyiidae | *Orseolia* | *Orseolia oryzae* | rice gall midges | Non-invasive | LC761943 |
| 21 | Insecta | Diptera | Cecidomyiidae | *Orseolia* | *Orseolia oryzivora* | rice gall midges | Non-invasive | KP109819 |
| 22 | Insecta | Diptera | Chironomidae | *Apedilum* | *Apedilum subcinctum* | root/stem feeders | Non-invasive | / |
| 23 | Insecta | Diptera | Chironomidae | *Cricotopus* | *Cricotopus sylvestris* | root/stem feeders | Non-invasive | OQ699201 |
| 24 | Insecta | Diptera | Chloropidae | *Chlorops* | *Chlorops oryzae* | root/stem feeders | Non-invasive | MW438309 |
| 25 | Insecta | Diptera | Diopsidae | *Diopsis* | *Diopsis apicalis* | stem borers | Non-invasive | / |
| 26 | Insecta | Diptera | Diopsidae | *Diopsis* | *Diopsis macrophthalma* | stem borers | Non-invasive | / |
| 27 | Insecta | Diptera | Ephydridae | *Hydrellia* | *Hydrellia griseola* | foliage feeders | Non-invasive | MF059320 |
| 28 | Insecta | Diptera | Ephydridae | *Hydrellia* | *Hydrellia philippina* | foliage feeders | Non-invasive | / |
| 29 | Insecta | Diptera | Ephydridae | *Hydrellia* | *Hydrellia prosternalis* | foliage feeders | Non-invasive | / |
| 30 | Insecta | Diptera | Ephydridae | *Hydrellia* | *Hydrellia sasakii* | foliage feeders | Non-invasive | / |
| 31 | Insecta | Diptera | Ephydridae | *Hydrellia* | *Hydrellia wirthi* | foliage feeders | Invasive | / |
| 32 | Insecta | Diptera | Muscidae | *Atherigona* | *Atherigona exigua* | root/stem feeders | Non-invasive | / |
| 33 | Insecta | Diptera | Muscidae | *Atherigona* | *Atherigona oryzae* | root/stem feeders | Non-invasive | JN298781 |
| 34 | Insecta | Hemiptera | Aleyrodidae | *Aleurocybotus* | *Aleurocybotus occiduus* | foliage feeders | Invasive | / |
| 35 | Insecta | Hemiptera | Aleyrodidae | *Vasdavidius* | *Vasdavidius indicus* | foliage feeders | Non-invasive | ON615604 |

| ID | Class | Order | Family | Genus | Species | Feeder type | Invasion status | Accession Numbers |
|---|---|---|---|---|---|---|---|---|
| 36 | Insecta | Hemiptera | Alydidae | *Leptocorisa* | *Leptocorisa acuta* | panicle feeders | Non-invasive | OL697744 |
| 37 | Insecta | Hemiptera | Alydidae | *Leptocorisa* | *Leptocorisa chinensis* | panicle feeders | Non-invasive | NC_061737 |
| 38 | Insecta | Hemiptera | Alydidae | *Leptocorisa* | *Leptocorisa oratoria* | panicle feeders | Non-invasive | OL697746 |
| 39 | Insecta | Hemiptera | Aphididae | *Hysteroneura* | *Hysteroneura setariae* | foliage feeders | Invasive | MN388830 |
| 40 | Insecta | Hemiptera | Aphididae | *Rhopalosiphum* | *Rhopalosiphum padi* | foliage feeders | Invasive | HQ528265 |
| 41 | Insecta | Hemiptera | Aphididae | *Rhopalosiphum* | *Rhopalosiphum rufiabdominale* | root/stem feeders | Invasive | NC_062327 |
| 42 | Insecta | Hemiptera | Aphididae | *Tetraneura* | *Tetraneura nigriabdominalis* | root/stem feeders | Invasive | KR573634 |
| 43 | Insecta | Hemiptera | Blissidae | *Blissus* | *Blissus leucopterus* | root/stem feeders | Invasive | KR041182 |
| 44 | Insecta | Hemiptera | Cercopidae | *Deois* | *Deois flavopicta* | leafhoppers | Non-invasive | KX239940 |
| 45 | Insecta | Hemiptera | Cercopidae | *Locris* | *Locris maculata* | leafhoppers | Non-invasive | NC_021100 |
| 46 | Insecta | Hemiptera | Cercopidae | *Locris* | *Locris rubra* | leafhoppers | Non-invasive | JF821187 |
| 47 | Insecta | Hemiptera | Cicadellidae | *Cofana* | *Cofana spectra* | leafhoppers | Non-invasive | PP968141 |
| 48 | Insecta | Hemiptera | Cicadellidae | *Cofana* | *Cofana unimaculata* | leafhoppers | Non-invasive | MW644814 |
| 49 | Insecta | Hemiptera | Cicadellidae | *Hortensia* | *Hortensia similis* | leafhoppers | Non-invasive | MW558141 |
| 50 | Insecta | Hemiptera | Cicadellidae | *Maiestas* | *Maiestas dorsalis* | leafhoppers | Non-invasive | KX786285 |
| 51 | Insecta | Hemiptera | Cicadellidae | *Nephotettix* | *Nephotettix afer* | leafhoppers | Non-invasive | / |
| 52 | Insecta | Hemiptera | Cicadellidae | *Nephotettix* | *Nephotettix cincticeps* | leafhoppers | Non-invasive | KP749836 |
| 53 | Insecta | Hemiptera | Cicadellidae | *Nephotettix* | *Nephotettix malayanus* | leafhoppers | Non-invasive | NC_066981 |

| ID | Class | Order | Family | Genus | Species | Feeder type | Invasion status | Accession Numbers |
|---|---|---|---|---|---|---|---|---|
| 54 | Insecta | Hemiptera | Cicadellidae | *Nephotettix* | *Nephotettix modulatus* | leafhoppers | Non-invasive | LC775123 |
| 55 | Insecta | Hemiptera | Cicadellidae | *Nephotettix* | *Nephotettix nigropictus* | leafhoppers | Non-invasive | OK105080 |
| 56 | Insecta | Hemiptera | Cicadellidae | *Nephotettix* | *Nephotettix parvus* | leafhoppers | Non-invasive | OK105074 |
| 57 | Insecta | Hemiptera | Cicadellidae | *Nephotettix* | *Nephotettix virescens* | leafhoppers | Non-invasive | NC_066983 |
| 58 | Insecta | Hemiptera | Delphacidae | *Laodelphax* | *Laodelphax striatellus* | planthoppers | Non-invasive | JX880068 |
| 59 | Insecta | Hemiptera | Delphacidae | *Nilaparvata* | *Nilaparvata lugens* | planthoppers | Non-invasive | / |
| 60 | Insecta | Hemiptera | Delphacidae | *Sogatella* | *Sogatella furcifera* | planthoppers | Non-invasive | KC512915 |
| 61 | Insecta | Hemiptera | Delphacidae | *Tagosodes* | *Tagosodes cubanus* | planthoppers | Invasive | OM595595 |
| 62 | Insecta | Hemiptera | Delphacidae | *Tagosodes* | *Tagosodes orizicolus* | planthoppers | Invasive | / |
| 63 | Insecta | Hemiptera | Miridae | *Stenotus* | *Stenotus rubrovittatus* | panicle feeders | Non-invasive | AB518925 |
| 64 | Insecta | Hemiptera | Miridae | *Trigonotylus* | *Trigonotylus caelestialium* | panicle feeders | Non-invasive | AB646366 |
| 65 | Insecta | Hemiptera | Pentatomidae | *Eysarcoris* | *Eysarcoris ventralis* | panicle feeders | Invasive | ON041148 |
| 66 | Insecta | Hemiptera | Pentatomidae | *Nezara* | *Nezara viridula* | panicle feeders | Invasive | / |
| 67 | Insecta | Hemiptera | Pentatomidae | *Niphe* | *Niphe elongata* | panicle feeders | Non-invasive | KC155923 |
| 68 | Insecta | Hemiptera | Pentatomidae | *Oebalus* | *Oebalus insularis* | panicle feeders | Invasive | / |
| 69 | Insecta | Hemiptera | Pentatomidae | *Oebalus* | *Oebalus poecilus* | panicle feeders | Non-invasive | JQ218463 |
| 70 | Insecta | Hemiptera | Pentatomidae | *Oebalus* | *Oebalus pugnax* | panicle feeders | Non-invasive | HM374566 |
| 71 | Insecta | Hemiptera | Pentatomidae | *Oebalus* | *Oebalus ypsilongriseus* | panicle feeders | Invasive | / |
| 72 | Insecta | Hemiptera | Pentatomidae | *Scotinophara* | *Scotinophara coarctata* | root/stem feeders | Invasive | / |
| 73 | Insecta | Hemiptera | Pentatomidae | *Scotinophara* | *Scotinophara latiuscula* | root/stem feeders | Non-invasive | / |

| ID | Class | Order | Family | Genus | Species | Feeder type | Invasion status | Accession Numbers |
|---|---|---|---|---|---|---|---|---|
| 74 | Insecta | Hemiptera | Pentatomidae | *Scotinophara* | *Scotinophara lurida* | root/stem feeders | Non-invasive | MF497733 |
| 75 | Insecta | Hemiptera | Pentatomidae | *Tibraca* | *Tibraca limbativentris* | root/stem feeders | Invasive | MW982717 |
| 76 | Insecta | Hemiptera | Pseudococcidae | *Brevennia* | *Brevennia rehi* | root/stem feeders | Invasive | / |
| 77 | Insecta | Lepidoptera | Crambidae | *Chilo* | *Chilo auricilia* | stem borers | Non-invasive | / |
| 78 | Insecta | Lepidoptera | Crambidae | *Chilo* | *Chilo partellus* | stem borers | Invasive | OQ154269 |
| 79 | Insecta | Lepidoptera | Crambidae | *Chilo* | *Chilo plejadellus* | stem borers | Non-invasive | LMDH170-11 |
| 80 | Insecta | Lepidoptera | Crambidae | *Chilo* | *Chilo polychrysa* | stem borers | Non-invasive | MW559555 |
| 81 | Insecta | Lepidoptera | Crambidae | *Chilo* | *Chilo suppressalis* | stem borers | Invasive | JF339041 |
| 82 | Insecta | Lepidoptera | Crambidae | *Chilo* | *Chilo zacconius* | stem borers | Non-invasive | / |
| 83 | Insecta | Lepidoptera | Crambidae | *Cnaphalocrocis* | *Cnaphalocrocis medinalis* | foliage feeders | Non-invasive | NC_015985 |
| 84 | Insecta | Lepidoptera | Crambidae | *Cnaphalocrocis* | *Cnaphalocrocis patnalis* | foliage feeders | Non-invasive | NC_060868 |
| 85 | Insecta | Lepidoptera | Crambidae | *Cnaphalocrocis* | *Cnaphalocrocis ruralis* | foliage feeders | Non-invasive | / |
| 86 | Insecta | Lepidoptera | Crambidae | *Cnaphalocrocis* | *Marasmia exigua* | foliage feeders | Non-invasive | MN877384 |
| 87 | Insecta | Lepidoptera | Crambidae | *Cnaphalocrocis* | *Marasmia trapezalis* | foliage feeders | Non-invasive | JX017849 |
| 88 | Insecta | Lepidoptera | Crambidae | *Diatraea* | *Diatraea saccharalis* | stem borers | Invasive | JN108964 |
| 89 | Insecta | Lepidoptera | Crambidae | *Eoreuma* | *Eoreuma loftini* | stem borers | Invasive | KM068906 |
| 90 | Insecta | Lepidoptera | Crambidae | *Parapoynx* | *Parapoynx fluctuosalis* | foliage feeders | Invasive | OR501826 |
| 91 | Insecta | Lepidoptera | Crambidae | *Parapoynx* | *Parapoynx stagnalis* | foliage feeders | Non-invasive | MT357095 |
| 92 | Insecta | Lepidoptera | Crambidae | *Rupela* | *Rupela albina* | stem borers | Non-invasive | MK612285 |
| 93 | Insecta | Lepidoptera | Crambidae | *Scirpophaga* | *Scirpophaga incertulas* | stem borers | Non-invasive | KF751706 |

| ID | Class | Order | Family | Genus | Species | Feeder type | Invasion status | Accession Numbers |
|---|---|---|---|---|---|---|---|---|
| 94 | Insecta | Lepidoptera | Crambidae | *Scirpophaga* | *Scirpophaga innotata* | stem borers | Non-invasive | AB495264 |
| 95 | Insecta | Lepidoptera | Erebidae | *Rivula* | *Rivula atimeta* | foliage feeders | Non-invasive | MK566630 |
| 96 | Insecta | Lepidoptera | Hesperiidae | *Parnara* | *Parnara guttatus* | foliage feeders | Non-invasive | JX101619 |
| 97 | Insecta | Lepidoptera | Hesperiidae | *Pelopidas* | *Pelopidas mathias* | foliage feeders | Non-invasive | MW264491 |
| 98 | Insecta | Lepidoptera | Noctuidae | *Mythimna* | *Mythimna separata* | foliage feeders | Non-invasive | KM099034 |
| 99 | Insecta | Lepidoptera | Noctuidae | *Mythimna* | *Mythimna unipuncta* | foliage feeders | Invasive | KX281211 |
| 100 | Insecta | Lepidoptera | Noctuidae | *Naranga* | *Naranga aenescens* | foliage feeders | Invasive | / |
| 101 | Insecta | Lepidoptera | Noctuidae | *Sesamia* | *Sesamia calamistis* | stem borers | Non-invasive | KF972231 |
| 102 | Insecta | Lepidoptera | Noctuidae | *Sesamia* | *Sesamia inferens* | stem borers | Non-invasive | JN039362 |
| 103 | Insecta | Lepidoptera | Noctuidae | *Sesamia* | *Sesamia nonagrioides* | stem borers | Non-invasive | JF274172 |
| 104 | Insecta | Lepidoptera | Noctuidae | *Spodoptera* | *Spodoptera exempta* | foliage feeders | Invasive | NC_054179 |
| 105 | Insecta | Lepidoptera | Noctuidae | *Spodoptera* | *Spodoptera frugiperda* | foliage feeders | Invasive | HQ177347 |
| 106 | Insecta | Lepidoptera | Noctuidae | *Spodoptera* | *Spodoptera litura* | foliage feeders | Invasive | JQ064568 |
| 107 | Insecta | Lepidoptera | Noctuidae | *Spodoptera* | *Spodoptera mauritia* | foliage feeders | Invasive | MW666001 |
| 108 | Insecta | Lepidoptera | Nymphalidae | *Melanitis* | *Melanitis leda* | foliage feeders | Non-invasive | OL504684 |
| 109 | Insecta | Lepidoptera | Pyralidae | *Elasmopalpus* | *Elasmopalpus lignosella* | stem borers | Invasive | / |
| 110 | Insecta | Lepidoptera | Pyralidae | *Maliarpha* | *Maliarpha separatella* | stem borers | Non-invasive | BETAM750-17 |
| 111 | Insecta | Orthoptera | Acrididae | *Acrida* | *Acrida exaltata* | foliage feeders | Non-invasive | MW147492 |
| 112 | Insecta | Orthoptera | Acrididae | *Hieroglyphus* | *Hieroglyphus banian* | foliage feeders | Non-invasive | MZ695309 |
| 113 | Insecta | Orthoptera | Acrididae | *Hieroglyphus* | *Hieroglyphus daganensis* | foliage feeders | Non-invasive | / |

| ID | Class | Order | Family | Genus | Species | Feeder type | Invasion status | Accession Numbers |
|---|---|---|---|---|---|---|---|---|
| 114 | Insecta | Orthoptera | Acrididae | *Hieroglyphus* | *Hieroglyphus nigrorepletus* | foliage feeders | Non-invasive | JF838482 |
| 115 | Insecta | Orthoptera | Acrididae | *Locusta* | *Locusta migratoria* | foliage feeders | Non-invasive | NC_014891 |
| 116 | Insecta | Orthoptera | Acrididae | *Oedaleus* | *Oedaleus abruptus* | foliage feeders | Invasive | MK352098 |
| 117 | Insecta | Orthoptera | Acrididae | *Oedaleus* | *Oedaleus senegalensis* | foliage feeders | Non-invasive | / |
| 118 | Insecta | Orthoptera | Acrididae | *Oxya* | *Oxya chinensis* | foliage feeders | Invasive | NC_010219 |
| 119 | Insecta | Orthoptera | Acrididae | *Oxya* | *Oxya hyla* | foliage feeders | Non-invasive | KP313875 |
| 120 | Insecta | Orthoptera | Acrididae | *Oxya* | *Oxya japonica* | foliage feeders | Invasive | NC_043773 |
| 121 | Insecta | Orthoptera | Acrididae | *Schistocerca* | *Schistocerca gregaria* | foliage feeders | Non-invasive | NC_013240 |
| 122 | Insecta | Orthoptera | Gryllotalpidae | *Gryllotalpa* | *Gryllotalpa africana* | root/stem feeders | Non-invasive | OR974770 |
| 123 | Insecta | Orthoptera | Gryllotalpidae | *Gryllotalpa* | *Gryllotalpa orientalis* | root/stem feeders | Invasive | AY660929 |
| 124 | Insecta | Orthoptera | Gryllotalpidae | *Neocurtilla* | *Neocurtilla hexadactyla* | root/stem feeders | Invasive | / |
| 125 | Insecta | Orthoptera | Gryllotalpidae | *Neoscapteriscus* | *Neoscapteriscus didactylus* | root/stem feeders | Invasive | / |
| 126 | Insecta | Orthoptera | Pyrgomorphidae | *Zonocerus* | *Zonocerus variegatus* | foliage feeders | Non-invasive | MT011541 |
| 127 | Insecta | Thysanoptera | Phlaeothripidae | *Haplothrips* | *Haplothrips aculeatus* | panicle feeders | Non-invasive | MF716898 |
| 128 | Insecta | Thysanoptera | Phlaeothripidae | *Haplothrips* | *Haplothrips ganglbaueri* | panicle feeders | Non-invasive | OK668387 |
| 129 | Insecta | Thysanoptera | Thripidae | *Stenchaetothrips* | *Stenchaetothrips biformis* | foliage feeders | Invasive | OP913446 |

Note: All COI sequences listed in this table were retrieved from GenBank, except that of *Maliarpha separatella*, which was obtained from the BOLD Systems database.

**Table S2. The composition of the functional trait dataset of rice pests.** References used as sources for trait definition and inclusion in the invasiveness analysis.

| Functional traits | Type | Unit | Reference |
|---|---|---|---|
| Body length | continuous | mm | [1,2] |
| Fecundity | continuous | eggs/female | [2,3] |
| Host number | continuous | count | [1,2,4] |
| Voltinism | continuous | generations/year | [1,2] |
| Age at maturity | continuous | days | [2,5] |
| Lifespan | continuous | days | [2] |
| Migration distance | continuous | km | [6] |
| Oviposition substrate | categorical | plant/others (1/0) | [1] |
| Reproductive mode | categorical | asexual reproduction/others (1/0) | [4] |
| Habitat type | categorical | terrestrial/others (1/0) | [2] |

Reference list:


1. Helen F. Nahrung; Swain, A.J. Strangers in a Strange Land: Do Life History Traits Differ for Alien and Native Colonisers of Novel Environments? *Biol. Invasions* **2015**, *17*, 699–709, doi:10.1007/s10530-014-0761-7.
2. Zhao, Z.; Hui, C.; Peng, S.; Yi, S.; Li, Z.; Reddy, G.V.P.; Van Kleunen, M. The World's 100 Worst Invasive Alien Insect Species Differ in Their Characteristics from Related Non-invasive Species. *J. Appl. Ecol.* **2023**, *60*, 1929–1938, doi:10.1111/1365-2664.14485.
3. Liu, C.; Comte, L.; Olden, J.D. Heads You Win, Tails You Lose: Life-history Traits Predict Invasion and Extinction Risk of the World's Freshwater Fishes. *Aquatic Conservation* **2017**, *27*, 773–779, doi:10.1002/aqc.2740.
4. Mondor, E.B.; Tremblay, M.N.; Messing, R.H. Morphological and Ecological Traits Promoting Aphid Colonization of the Hawaiian Islands. *Biol. Invasions* **2006**, *9*, 87–100, doi:10.1007/s10530-006-9010-z.
5. Allen, W.L.; Street, S.E.; Capellini, I. Fast Life History Traits Promote Invasion Success in Amphibians and Reptiles. *Ecol. Lett.* **2017**, *20*, 222–230, doi:10.1111/ele.12728.
6. Soares, A.O.; Honěk, A.; Martinkova, Z.; Brown, P.M.J.; Borges, I. Can Native Geographical Range, Dispersal Ability and Development Rates Predict the Successful Establishment of Alien Ladybird (Coleoptera: Coccinellidae) Species in Europe? *Front. Ecol. Evol.* **2018**, *6*, 57, doi:10.3389/fevo.2018.00057.

**Table S3. Akaike's Information Criterion of models for phylogenetic imputation.**

| Model | AIC | Delta_AIC | Weight |
|---|---|---|---|
| Pagel's λ | 228.93 | 0.00 | 1.00 |
| Ornstein-Uhlenbeck | 383.00 | 154.06 | 0.00 |
| Brownian motion | 393.18 | 164.25 | 0.00 |
| Early-Burst | 395.18 | 166.25 | 0.00 |

Note: AIC represents Akaike's Information Criterion, where lower values indicate a better model fit. Delta AIC is the difference in AIC between a given model and the best-performing model (Pagel's λ, Delta_AIC = 0). Weight refers to the Akaike weight, representing the relative probability that a specific model is the best among the candidate set.

**Table S4. The functional trait dataset of global major rice pests.**

| ID | species | Body length | Fecundity | Host number | Voltinism | Age at maturity | Lifespan | Migration distance | Oviposition substrate | Reproductive mode | Habitat type |
|---|---|---|---|---|---|---|---|---|---|---|---|
| 1 | *Steneotarsonemus spinki* | 0.56 | 5.42 | 1.35 | 4.78 | 2.89 | 4.12 | 1.79 | 1 | 1 | 0 |
| 2 | *Oligonychus oryzae* | 0.59 | 3.64 | 1.00 | 3.52 | 2.97 | 3.90 | 1.79 | 1 | 1 | 0 |
| 3 | *Oligonychus pratensis* | 0.69 | 3.51 | 1.35 | 3.24 | 3.38 | 4.05 | 1.79 | 1 | 1 | 0 |
| 4 | *Afroryzophilus djibai* | 1.51 | 5.62 | 1.35 | 1.49 | 6.25 | 8.65 | 2.40 | 1 | 0 | 1 |
| 5 | *Echinocnemus oryzae* | 2.28 | 5.52 | 1.00 | 1.35 | 12.68 | 12.03 | 2.40 | 0 | 0 | 1 |
| 6 | *Echinocnemus squameus* | 2.01 | 4.38 | 1.00 | 1.19 | 8.71 | 10.78 | 2.40 | 0 | 0 | 1 |
| 7 | *Hydronomidius molitor* | 1.92 | 5.49 | 1.00 | 1.00 | 12.41 | 13.53 | 2.40 | 0 | 0 | 1 |
| 8 | *Lissorhoptrus brevirostris* | 1.63 | 5.85 | 2.01 | 1.19 | 5.47 | 9.96 | 2.40 | 1 | 0 | 1 |
| 9 | *Lissorhoptrus oryzophilus* | 1.58 | 6.52 | 3.33 | 1.49 | 5.12 | 11.54 | 4.62 | 1 | 1 | 1 |
| 10 | *Oryzophagus oryzae* | 1.64 | 6.54 | 1.35 | 1.61 | 5.40 | 8.08 | 2.40 | 1 | 0 | 1 |
| 11 | *Chaetocnema pulla* | 0.50 | 4.51 | 1.00 | 2.57 | 2.81 | 5.19 | 2.40 | 0 | 0 | 0 |
| 12 | *Dicladispa armigera* | 1.87 | 5.90 | 3.67 | 1.92 | 3.90 | 8.50 | 5.30 | 1 | 0 | 0 |
| 13 | *Leptispa pygmaea* | 2.26 | 3.45 | 1.35 | 1.60 | 3.70 | 6.23 | 2.40 | 1 | 0 | 0 |
| 14 | *Oulema oryzae* | 1.87 | 5.22 | 1.00 | 1.00 | 4.68 | 12.89 | 2.40 | 1 | 0 | 0 |
| 15 | *Trichispa sericea* | 1.72 | 7.39 | 1.00 | 1.72 | 4.94 | 7.94 | 2.40 | 1 | 0 | 0 |
| 16 | *Chnootriba similis* | 2.18 | 13.95 | 2.72 | 1.35 | 5.07 | 7.54 | 3.43 | 1 | 0 | 0 |
| 17 | *Holotrichia serrata* | 3.98 | 5.70 | 4.18 | 0.88 | 9.41 | 11.69 | 3.43 | 0 | 0 | 0 |

| ID | species | Body length | Fecundity | Host number | Voltinism | Age at maturity | Lifespan | Migration distance | Oviposition substrate | Reproductive mode | Habitat type |
|---|---|---|---|---|---|---|---|---|---|---|---|
| 18 | *Leucopholis irrorata* | 4.29 | 5.70 | 1.83 | 0.88 | 13.47 | 13.73 | 3.43 | 0 | 0 | 0 |
| 19 | *Leucopholis lepidophora* | 4.80 | 4.30 | 2.01 | 0.74 | 13.12 | 13.46 | 3.43 | 0 | 0 | 0 |
| 20 | *Orseolia oryzae* | 1.86 | 9.82 | 1.35 | 2.53 | 3.90 | 4.03 | 2.40 | 1 | 0 | 0 |
| 21 | *Orseolia oryzivora* | 1.86 | 11.00 | 1.35 | 2.60 | 3.80 | 4.02 | 2.40 | 1 | 0 | 0 |
| 22 | *Apedilum subcinctum* | 1.45 | 8.01 | 1.61 | 1.72 | 2.99 | 4.23 | 2.40 | 0 | 0 | 1 |
| 23 | *Cricotopus sylvestris* | 2.05 | 11.00 | 1.00 | 1.72 | 4.01 | 5.08 | 2.40 | 0 | 0 | 1 |
| 24 | *Chlorops oryzae* | 1.50 | 6.52 | 2.47 | 1.72 | 6.35 | 6.69 | 2.40 | 1 | 0 | 0 |
| 25 | *Diopsis apicalis* | 2.72 | 4.38 | 1.35 | 1.35 | 3.33 | 4.38 | 2.40 | 1 | 0 | 0 |
| 26 | *Diopsis macrophthalma* | 2.72 | 4.65 | 1.00 | 1.49 | 5.10 | 5.77 | 2.40 | 1 | 0 | 0 |
| 27 | *Hydrellia griseola* | 1.30 | 6.52 | 2.18 | 2.25 | 3.79 | 7.75 | 2.40 | 1 | 0 | 1 |
| 28 | *Hydrellia philippina* | 1.40 | 7.39 | 2.72 | 2.60 | 3.94 | 4.28 | 2.40 | 1 | 0 | 1 |
| 29 | *Hydrellia prosternalis* | 1.42 | 5.43 | 1.61 | 1.35 | 4.41 | 5.47 | 2.40 | 1 | 0 | 1 |
| 30 | *Hydrellia sasakii* | 1.37 | 3.98 | 1.00 | 2.18 | 4.02 | 4.51 | 2.40 | 1 | 0 | 1 |
| 31 | *Hydrellia wirthi* | 1.30 | 5.76 | 1.61 | 2.09 | 3.88 | 5.07 | 2.40 | 1 | 0 | 1 |
| 32 | *Atherigona exigua* | 1.67 | 7.39 | 2.33 | 2.15 | 3.59 | 4.38 | 3.43 | 1 | 0 | 0 |
| 33 | *Atherigona oryzae* | 1.71 | 4.38 | 3.75 | 2.05 | 3.94 | 4.65 | 3.43 | 1 | 0 | 0 |
| 34 | *Aleurocybotus occiduus* | 1.10 | 8.00 | 2.47 | 2.22 | 6.21 | 5.90 | 2.40 | 1 | 0 | 0 |
| 35 | *Vasdavidius indicus* | 0.91 | 9.30 | 2.47 | 2.40 | 4.79 | 5.20 | 2.40 | 1 | 1 | 0 |
| 36 | *Leptocorisa acuta* | 3.33 | 11.00 | 3.15 | 1.49 | 4.64 | 6.40 | 3.43 | 1 | 0 | 0 |

| ID | species | Body length | Fecundity | Host number | Voltinism | Age at maturity | Lifespan | Migration distance | Oviposition substrate | Reproductive mode | Habitat type |
|---|---|---|---|---|---|---|---|---|---|---|---|
| 37 | *Leptocorisa chinensis* | 3.44 | 14.27 | 1.35 | 1.35 | 4.16 | 7.29 | 3.43 | 1 | 0 | 0 |
| 38 | *Leptocorisa oratoria* | 3.53 | 8.81 | 2.33 | 2.01 | 4.25 | 7.24 | 3.43 | 1 | 0 | 0 |
| 39 | *Hysteroneura setariae* | 1.19 | 5.07 | 2.72 | 3.10 | 2.63 | 3.99 | 2.40 | 1 | 1 | 0 |
| 40 | *Rhopalosiphum padi* | 1.21 | 3.87 | 4.44 | 2.89 | 2.68 | 4.47 | 5.71 | 1 | 1 | 0 |
| 41 | *Rhopalosiphum rufiabdominale* | 1.44 | 4.78 | 4.12 | 3.75 | 2.46 | 3.73 | 2.40 | 0 | 1 | 0 |
| 42 | *Tetraneura nigriabdominalis* | 1.31 | 4.96 | 3.90 | 2.47 | 3.54 | 4.63 | 2.40 | 0 | 1 | 0 |
| 43 | *Blissus leucopterus* | 1.83 | 13.12 | 3.42 | 1.49 | 5.12 | 7.47 | 2.40 | 1 | 0 | 0 |
| 44 | *Deois flavopicta* | 2.72 | 5.47 | 2.33 | 1.72 | 5.63 | 6.17 | 2.40 | 0 | 0 | 0 |
| 45 | *Locris maculata* | 2.78 | 7.07 | 1.00 | 1.56 | 5.52 | 6.66 | 3.43 | 1 | 0 | 0 |
| 46 | *Locris rubra* | 2.83 | 7.10 | 1.00 | 1.54 | 5.57 | 6.70 | 3.43 | 1 | 0 | 0 |
| 47 | *Cofana spectra* | 2.53 | 6.46 | 2.33 | 1.96 | 4.48 | 5.52 | 2.40 | 1 | 0 | 0 |
| 48 | *Cofana unimaculata* | 2.20 | 5.47 | 2.47 | 1.72 | 4.96 | 6.37 | 2.40 | 1 | 0 | 0 |
| 49 | *Hortensia similis* | 2.13 | 6.98 | 3.33 | 2.08 | 4.55 | 5.83 | 2.40 | 1 | 0 | 0 |
| 50 | *Maiestas dorsalis* | 1.83 | 7.02 | 2.47 | 2.40 | 4.35 | 5.20 | 3.43 | 1 | 0 | 0 |
| 51 | *Nephotettix afer* | 1.87 | 8.73 | 1.00 | 2.09 | 4.91 | 6.18 | 2.40 | 1 | 0 | 0 |
| 52 | *Nephotettix cincticeps* | 1.99 | 8.81 | 2.47 | 2.25 | 5.54 | 7.26 | 3.43 | 1 | 0 | 0 |
| 53 | *Nephotettix malayanus* | 1.89 | 11.00 | 2.01 | 2.53 | 4.05 | 5.24 | 2.40 | 1 | 0 | 0 |

| ID | species | Body length | Fecundity | Host number | Voltinism | Age at maturity | Lifespan | Migration distance | Oviposition substrate | Reproductive mode | Habitat type |
|---|---|---|---|---|---|---|---|---|---|---|---|
| 54 | *Nephotettix modulatus* | 1.93 | 10.26 | 1.00 | 2.43 | 4.69 | 5.92 | 2.40 | 1 | 0 | 0 |
| 55 | *Nephotettix nigropictus* | 2.01 | 9.40 | 3.15 | 2.47 | 5.43 | 7.02 | 2.40 | 1 | 0 | 0 |
| 56 | *Nephotettix parvus* | 1.85 | 10.51 | 1.35 | 1.61 | 5.08 | 6.73 | 2.40 | 1 | 0 | 0 |
| 57 | *Nephotettix virescens* | 1.98 | 11.12 | 1.00 | 2.66 | 4.60 | 5.81 | 3.43 | 1 | 0 | 0 |
| 58 | *Laodelphax striatellus* | 1.66 | 8.83 | 3.98 | 2.01 | 4.35 | 5.24 | 6.40 | 1 | 0 | 0 |
| 59 | *Nilaparvata lugens* | 1.97 | 9.42 | 2.01 | 2.33 | 3.79 | 4.63 | 6.22 | 1 | 0 | 0 |
| 60 | *Sogatella furcifera* | 1.77 | 9.70 | 2.33 | 2.72 | 4.13 | 5.05 | 6.91 | 1 | 0 | 0 |
| 61 | *Tagosodes cubanus* | 1.35 | 9.76 | 1.35 | 2.30 | 3.12 | 3.93 | 2.40 | 1 | 0 | 0 |
| 62 | *Tagosodes orizicolus* | 1.55 | 11.10 | 1.00 | 1.49 | 3.67 | 5.44 | 3.43 | 1 | 0 | 0 |
| 63 | *Stenotus rubrovittatus* | 1.92 | 8.24 | 1.35 | 1.61 | 3.15 | 5.12 | 2.40 | 1 | 0 | 0 |
| 64 | *Trigonotylus caelestialium* | 2.00 | 7.63 | 1.83 | 1.49 | 3.63 | 4.99 | 2.40 | 1 | 0 | 0 |
| 65 | *Eysarcoris ventralis* | 2.13 | 8.28 | 1.35 | 1.72 | 5.22 | 7.08 | 2.40 | 1 | 0 | 0 |
| 66 | *Nezara viridula* | 3.01 | 8.17 | 7.16 | 1.83 | 5.70 | 7.82 | 4.62 | 1 | 0 | 0 |
| 67 | *Niphe elongata* | 3.03 | 6.99 | 1.00 | 1.35 | 6.67 | 8.31 | 2.40 | 1 | 0 | 0 |
| 68 | *Oebalus insularis* | 2.60 | 11.00 | 1.35 | 1.71 | 3.62 | 5.55 | 2.40 | 1 | 0 | 0 |
| 69 | *Oebalus poecilus* | 2.53 | 12.69 | 1.35 | 1.49 | 5.12 | 5.88 | 2.40 | 1 | 0 | 0 |
| 70 | *Oebalus pugnax* | 2.72 | 14.78 | 2.18 | 1.72 | 4.22 | 6.84 | 2.40 | 1 | 0 | 0 |
| 71 | *Oebalus ypsilongriseus* | 2.63 | 10.89 | 1.35 | 1.64 | 4.39 | 6.15 | 2.40 | 1 | 0 | 0 |

| ID | species | Body length | Fecundity | Host number | Voltinism | Age at maturity | Lifespan | Migration distance | Oviposition substrate | Reproductive mode | Habitat type |
|---|---|---|---|---|---|---|---|---|---|---|---|
| 72 | *Scotinophara coarctata* | 2.47 | 7.95 | 2.18 | 1.35 | 5.25 | 10.35 | 3.43 | 1 | 0 | 0 |
| 73 | *Scotinophara latiuscula* | 2.49 | 6.41 | 1.43 | 1.19 | 5.22 | 10.21 | 3.43 | 1 | 0 | 0 |
| 74 | *Scotinophara lurida* | 2.45 | 8.81 | 1.00 | 1.49 | 5.20 | 10.89 | 3.43 | 1 | 0 | 0 |
| 75 | *Tibraca limbativentris* | 3.19 | 16.09 | 4.05 | 1.72 | 5.83 | 7.95 | 3.43 | 1 | 0 | 0 |
| 76 | *Brevennia rehi* | 1.51 | 9.33 | 3.42 | 2.94 | 4.29 | 4.60 | 1.79 | 1 | 1 | 0 |
| 77 | *Chilo auricilia* | 2.60 | 9.82 | 2.01 | 2.01 | 5.02 | 5.26 | 3.43 | 1 | 0 | 0 |
| 78 | *Chilo partellus* | 3.16 | 13.12 | 4.05 | 1.61 | 5.35 | 5.71 | 5.30 | 1 | 0 | 0 |
| 79 | *Chilo plejadellus* | 4.05 | 10.68 | 1.00 | 1.49 | 6.21 | 6.20 | 3.43 | 1 | 0 | 0 |
| 80 | *Chilo polychrysa* | 2.89 | 11.00 | 2.18 | 2.60 | 5.02 | 5.20 | 3.43 | 1 | 0 | 0 |
| 81 | *Chilo suppressalis* | 3.21 | 12.33 | 3.75 | 1.72 | 5.47 | 5.72 | 4.62 | 1 | 0 | 0 |
| 82 | *Chilo zacconius* | 2.83 | 10.81 | 1.35 | 2.18 | 5.54 | 5.83 | 3.43 | 1 | 0 | 0 |
| 83 | *Cnaphalocrocis medinalis* | 2.47 | 8.14 | 3.42 | 2.18 | 4.54 | 4.99 | 6.40 | 1 | 0 | 0 |
| 84 | *Cnaphalocrocis patnalis* | 2.83 | 8.00 | 3.51 | 1.94 | 4.91 | 5.22 | 3.43 | 1 | 0 | 0 |
| 85 | *Cnaphalocrocis ruralis* | 2.83 | 7.17 | 1.00 | 1.73 | 4.53 | 5.01 | 3.43 | 1 | 0 | 0 |
| 86 | *Marasmia exigua* | 2.53 | 7.92 | 3.42 | 1.83 | 4.80 | 5.10 | 3.43 | 1 | 0 | 0 |
| 87 | *Marasmia trapezalis* | 2.78 | 6.85 | 2.60 | 1.93 | 4.15 | 4.65 | 3.43 | 1 | 0 | 0 |
| 88 | *Diatraea saccharalis* | 3.69 | 15.64 | 2.60 | 2.01 | 5.47 | 5.72 | 3.43 | 1 | 0 | 0 |
| 89 | *Eoreuma loftini* | 2.94 | 12.41 | 2.94 | 2.01 | 5.96 | 6.37 | 3.43 | 1 | 0 | 0 |
| 90 | *Parapoynx fluctuosalis* | 2.25 | 8.14 | 1.00 | 1.35 | 5.16 | 5.35 | 2.40 | 0 | 0 | 1 |

| ID | species | Body length | Fecundity | Host number | Voltinism | Age at maturity | Lifespan | Migration distance | Oviposition substrate | Reproductive mode | Habitat type |
|---|---|---|---|---|---|---|---|---|---|---|---|
| 91 | *Parapoynx stagnalis* | 2.94 | 7.55 | 2.47 | 1.92 | 4.50 | 4.65 | 3.43 | 1 | 0 | 0 |
| 92 | *Rupela albina* | 2.73 | 6.09 | 1.35 | 1.35 | 5.94 | 6.21 | 3.43 | 1 | 0 | 0 |
| 93 | *Scirpophaga incertulas* | 2.71 | 8.84 | 1.00 | 1.92 | 6.31 | 6.44 | 4.62 | 1 | 0 | 0 |
| 94 | *Scirpophaga innotata* | 2.99 | 8.84 | 1.83 | 2.18 | 6.14 | 6.59 | 3.43 | 1 | 0 | 0 |
| 95 | *Rivula atimeta* | 2.20 | 8.28 | 1.61 | 2.20 | 3.87 | 4.15 | 3.43 | 1 | 0 | 0 |
| 96 | *Parnara guttatus* | 3.41 | 10.84 | 2.94 | 2.01 | 5.10 | 5.62 | 6.22 | 1 | 0 | 0 |
| 97 | *Pelopidas mathias* | 3.54 | 6.05 | 3.98 | 1.61 | 4.87 | 5.21 | 5.71 | 1 | 0 | 0 |
| 98 | *Mythimna separata* | 3.47 | 17.20 | 4.57 | 2.01 | 4.99 | 5.59 | 7.31 | 1 | 0 | 0 |
| 99 | *Mythimna unipuncta* | 3.67 | 19.64 | 3.83 | 1.83 | 5.68 | 6.25 | 5.71 | 1 | 0 | 0 |
| 100 | *Naranga aenescens* | 2.40 | 11.06 | 2.60 | 1.92 | 4.71 | 4.85 | 3.43 | 1 | 0 | 0 |
| 101 | *Sesamia calamistis* | 3.87 | 13.57 | 2.83 | 1.92 | 6.83 | 7.18 | 4.62 | 1 | 0 | 0 |
| 102 | *Sesamia inferens* | 3.15 | 10.98 | 4.57 | 2.01 | 6.41 | 6.63 | 4.62 | 1 | 0 | 0 |
| 103 | *Sesamia nonagrioides* | 3.33 | 12.41 | 3.59 | 1.61 | 6.00 | 6.37 | 4.62 | 1 | 0 | 0 |
| 104 | *Spodoptera exempta* | 3.33 | 18.23 | 3.83 | 2.53 | 4.41 | 5.02 | 5.71 | 1 | 0 | 0 |
| 105 | *Spodoptera frugiperda* | 3.32 | 20.22 | 10.05 | 1.92 | 5.32 | 6.00 | 6.22 | 1 | 0 | 0 |
| 106 | *Spodoptera litura* | 3.42 | 20.51 | 8.58 | 2.40 | 4.78 | 5.58 | 5.71 | 1 | 0 | 0 |
| 107 | *Spodoptera mauritia* | 3.42 | 20.09 | 4.25 | 2.01 | 4.91 | 5.68 | 4.62 | 1 | 0 | 0 |
| 108 | *Melanitis leda* | 3.83 | 6.52 | 3.90 | 1.72 | 4.68 | 5.56 | 3.43 | 1 | 0 | 0 |
| 109 | *Elasmopalpus lignosella* | 2.63 | 11.42 | 4.05 | 1.49 | 5.04 | 5.54 | 3.43 | 0 | 0 | 0 |

| ID | species | Body length | Fecundity | Host number | Voltinism | Age at maturity | Lifespan | Migration distance | Oviposition substrate | Reproductive mode | Habitat type |
|---|---|---|---|---|---|---|---|---|---|---|---|
| 110 | Maliarpha separatella | 3.05 | 11.91 | 2.33 | 1.72 | 7.09 | 7.53 | 3.43 | 1 | 0 | 0 |
| 111 | Acrida exaltata | 5.30 | 9.45 | 1.83 | 1.35 | 6.95 | 7.70 | 3.43 | 0 | 0 | 0 |
| 112 | Hieroglyphus banian | 4.92 | 8.14 | 2.83 | 1.00 | 6.96 | 8.24 | 3.43 | 0 | 0 | 0 |
| 113 | Hieroglyphus daganensis | 5.10 | 7.51 | 1.35 | 1.00 | 6.99 | 8.37 | 3.43 | 0 | 0 | 0 |
| 114 | Hieroglyphus nigrorepletus | 4.79 | 7.04 | 1.61 | 1.00 | 6.35 | 7.87 | 3.43 | 0 | 0 | 0 |
| 115 | Locusta migratoria | 5.29 | 10.20 | 3.42 | 1.72 | 5.12 | 6.05 | 5.99 | 0 | 1 | 0 |
| 116 | Oedaleus abruptus | 3.41 | 7.66 | 1.00 | 1.61 | 5.79 | 6.61 | 3.43 | 0 | 0 | 0 |
| 117 | Oedaleus senegalensis | 4.68 | 6.71 | 3.51 | 1.61 | 4.38 | 5.10 | 5.86 | 0 | 0 | 0 |
| 118 | Oxya chinensis | 4.18 | 6.54 | 2.33 | 1.19 | 7.42 | 9.22 | 3.43 | 0 | 1 | 0 |
| 119 | Oxya hyla | 3.96 | 6.32 | 1.00 | 1.35 | 8.07 | 8.79 | 3.43 | 0 | 0 | 0 |
| 120 | Oxya japonica | 4.25 | 7.51 | 2.33 | 1.35 | 6.43 | 7.78 | 3.43 | 0 | 1 | 0 |
| 121 | Schistocerca gregaria | 5.61 | 9.93 | 7.85 | 1.72 | 5.74 | 8.00 | 8.01 | 0 | 1 | 0 |
| 122 | Gryllotalpa africana | 4.52 | 4.96 | 3.42 | 1.00 | 8.35 | 12.45 | 3.43 | 0 | 0 | 0 |
| 123 | Gryllotalpa orientalis | 4.47 | 7.39 | 3.42 | 0.88 | 12.06 | 15.61 | 3.43 | 0 | 0 | 0 |
| 124 | Neocurtilla hexadactyla | 4.12 | 5.37 | 2.01 | 0.88 | 12.41 | 15.46 | 3.43 | 0 | 0 | 0 |
| 125 | Neoscapteriscus didactylus | 4.27 | 6.17 | 2.47 | 1.19 | 11.37 | 15.02 | 3.43 | 0 | 0 | 0 |
| 126 | Zonocerus variegatus | 4.99 | 6.22 | 5.61 | 1.19 | 11.08 | 12.71 | 4.62 | 0 | 0 | 0 |

| ID | species | Body length | Fecundity | Host number | Voltinism | Age at maturity | Lifespan | Migration distance | Oviposition substrate | Reproductive mode | Habitat type |
|---|---|---|---|---|---|---|---|---|---|---|---|
| 127 | Haplothrips aculeatus | 1.31 | 3.47 | 4.91 | 2.78 | 3.67 | 5.22 | 2.40 | 1 | 1 | 0 |
| 128 | Haplothrips ganglbaueri | 1.19 | 2.83 | 1.83 | 2.62 | 3.38 | 4.38 | 2.40 | 1 | 1 | 0 |
| 129 | Stenchaetothrips biformis | 1.05 | 6.64 | 3.98 | 3.24 | 3.06 | 4.63 | 2.40 | 1 | 1 | 0 |

Note: All continuous variables have been log-transformed.

**Table S5. Parameter estimates for the Bayesian regression models.** See also Figure 4.

| Variables | Estimate | Est.Error | l-80% CI | u-80% CI | l-95% CI | u-95% CI | Rhat | Bulk_ESS | Tail_ESS | Bayesian model |
|---|---|---|---|---|---|---|---|---|---|---|
| Migration distance | -0.54 | 0.32 | -0.93 | -0.13 | -1.17 | 0.10 | 1.00 | 8890.29 | 6333.33 | PGLMM |
| Body length | -0.51 | 0.39 | -1.01 | -0.02 | -1.28 | 0.26 | 1.00 | 10516.43 | 6467.22 | PGLMM |
| Oviposition substrate | -0.26 | 0.47 | -0.85 | 0.33 | -1.17 | 0.68 | 1.00 | 10679.26 | 6035.68 | PGLMM |
| Age at maturity | -0.06 | 0.30 | -0.44 | 0.32 | -0.64 | 0.52 | 1.00 | 7874.83 | 5519.11 | PGLMM |
| Habitat type | 0.04 | 0.47 | -0.56 | 0.64 | -0.87 | 0.95 | 1.00 | 14372.30 | 5635.98 | PGLMM |
| Lifespan | 0.19 | 0.27 | -0.15 | 0.53 | -0.36 | 0.72 | 1.00 | 6972.11 | 5418.50 | PGLMM |
| Voltinism | 0.20 | 0.44 | -0.36 | 0.77 | -0.68 | 1.05 | 1.00 | 11126.57 | 6538.96 | PGLMM |
| Reproductive mode | 0.43 | 0.49 | -0.19 | 1.07 | -0.57 | 1.36 | 1.00 | 9495.16 | 5650.81 | PGLMM |
| Fecundity | 0.46 | 0.18 | 0.25 | 0.69 | 0.17 | 0.87 | 1.00 | 3355.14 | 3416.25 | PGLMM |
| Host number | 0.48 | 0.28 | 0.13 | 0.83 | -0.08 | 1.06 | 1.00 | 7273.29 | 5015.09 | PGLMM |
| Oviposition substrate | -0.48 | 0.41 | -1.01 | 0.05 | -1.27 | 0.36 | 1.00 | 8882.64 | 5560.85 | GLMM |
| Migration distance | -0.40 | 0.23 | -0.71 | -0.11 | -0.87 | 0.04 | 1.00 | 8029.19 | 6305.23 | GLMM |
| Body length | -0.36 | 0.28 | -0.72 | -0.01 | -0.91 | 0.17 | 1.00 | 7899.33 | 5535.39 | GLMM |
| Habitat type | -0.18 | 0.42 | -0.73 | 0.36 | -1.01 | 0.66 | 1.00 | 9407.75 | 5244.45 | GLMM |
| Age at maturity | -0.05 | 0.19 | -0.30 | 0.19 | -0.43 | 0.32 | 1.00 | 6994.53 | 6127.26 | GLMM |
| Lifespan | 0.19 | 0.16 | -0.01 | 0.39 | -0.12 | 0.50 | 1.00 | 6733.04 | 5902.37 | GLMM |
| Fecundity | 0.27 | 0.08 | 0.18 | 0.37 | 0.13 | 0.43 | 1.00 | 7654.46 | 6019.68 | GLMM |
| Voltinism | 0.38 | 0.36 | -0.08 | 0.84 | -0.32 | 1.08 | 1.00 | 8510.80 | 6088.10 | GLMM |

| Variables | Estimate | Est.Error | l-80% CI | u-80% CI | l-95% CI | u-95% CI | Rhat | Bulk_ESS | Tail_ESS | Bayesian model |
|---|---|---|---|---|---|---|---|---|---|---|
| Host number | 0.43 | 0.19 | 0.19 | 0.67 | 0.07 | 0.82 | 1.00 | 8489.84 | 5734.10 | GLMM |
| Reproductive mode | 0.78 | 0.43 | 0.24 | 1.32 | -0.05 | 1.61 | 1.00 | 8935.51 | 5941.00 | GLMM |

Note: Estimate represents the posterior mean of the regression coefficients, and Est.Error indicates the standard error. l-CI and u-CI denote the lower and upper bounds of the 80% and 95% credible intervals, respectively. Rhat is the Gelman-Rubin convergence diagnostic, where a value of 1.00 indicates successful model convergence across all Markov chains. Bulk_ESS and Tail_ESS refer to the bulk and tail effective sample sizes, indicating sufficient sampling efficiency.

**Table S6. Comparison of variance components between Bayesian PGLMM and Bayesian GLMM.**

| Model | Variance Component | Mean | Lower 95% CI | Upper 95% CI |
|---|---|---|---|---|
| PGLMM | Fixed effects | 0.22 | 0.06 | 0.39 |
| PGLMM | Phylogenetic random effect | 0.48 | 0.13 | 0.82 |
| PGLMM | Random effect (Feeding guilds) | 0.09 | 0.00 | 0.41 |
| PGLMM | Residual variance | 0.22 | 0.04 | 0.51 |
| PGLMM | Total variance | 0.78 | 0.49 | 0.96 |
| GLMM | Fixed effects | 0.30 | 0.14 | 0.47 |
| GLMM | Random effect (Feeding guilds) | 0.09 | 0.00 | 0.44 |
| GLMM | Residual variance | 0.61 | 0.37 | 0.79 |
| GLMM | Total variance | 0.39 | 0.21 | 0.63 |

Note: Mean represents the posterior mean of the estimated variance components. Lower 95% CI and Upper 95% CI denote the lower and upper bounds of the 95% credible intervals, respectively.

# Supporting Figures

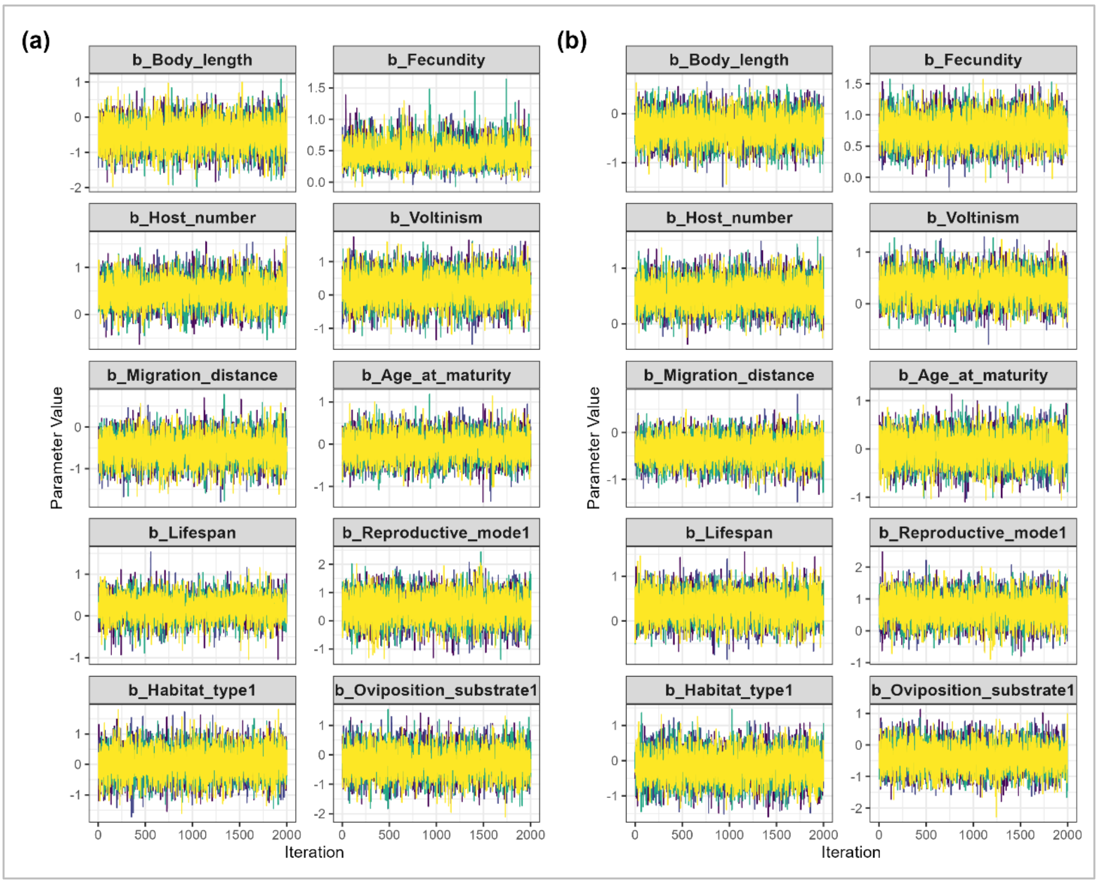


**Figure S1 MCMC sampling trajectory plot for fixed effects parameters in the Bayesian Models.** (a) Bayesian phylogenetic generalized linear mixed model. (b) Bayesian generalized linear mixed model. Posterior sampling records of regression coefficients (fixed effects) for the 10 functional traits used in the model. The horizontal axis represents the iteration count (2,000 iterations, excluding the warm-up phase), while the vertical axis shows the parameter estimates. Different colors denote four independent Markov chains. All parameters exhibit stable trajectories with well-mixed chains, indicating successful model convergence.

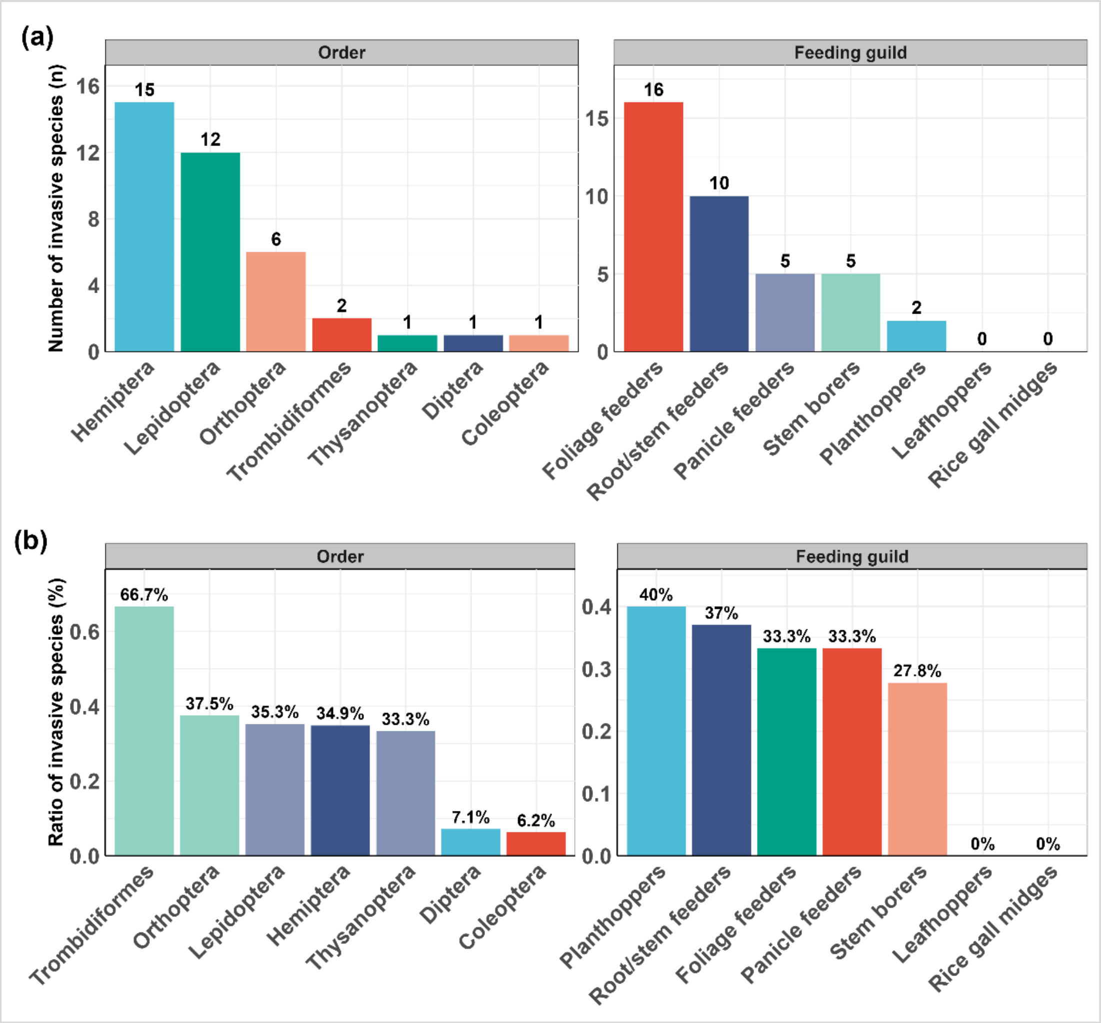


**Figure S2 Taxonomic and feeding guild patterns of invasive rice pests.** (a) Number of invasive species across taxonomic orders and feeding guilds among the 129 rice pest species included in this study. (b) Proportion of invasive species within each taxonomic order and feeding guild.
